# Urinary Disinfectants

**Published:** 1907-04-06

**Authors:** 


### Therapeutics.

### URINARY DISINFECTANTS.

Since the introduction of the drug commonly known as urotropine, the problem of urinary disinfection has been greatly simplified. It has long been realised that most conditions of cystitis, pyuria (bladder), and bacilluria, if they are not actually caused by the alkalinity of the urine, are very greatly favoured thereby, and that in an acid urine the processes do not flourish. At any rate, it was obvious to the clinical observer that when the urine became clear it usually also became acid. But the drugs hitherto administered in the hope of rendering the urine acid are not dependable or rapid in their action. Benzoic acid, sodium benzoate, and di-sodium phosphate are very capricious and cannot be taken for considerable periods without affecting the reaction of the urine. The converse process of rendering an acid urine alkaline is comparatively simple, but excepting in gonorrhœal urethritis and hypothetically in gout, rheumatism, and other forms of arthritis this is never required in treatment.

Disinfection by means of remedies which could be taken by the mouth instead of the laborious method of repeatedly washing out the bladder, was urgently desired, and at last, with the discovery of formic aldehyde as an antiseptic and bactericide, the new remedy was hit upon, empirically perhaps, but satisfactorily. For it is very doubtful whether the action of the new drugs depends really on formaldehyde liberated and exerting a specific bactericidal power, rather than on the production of a strongly acid reaction. We see an analogous illustration in the superiority of dilute solutions of nitric acid for irrigation of the bladder over all the more complex disinfectants whose mode of action is less intelligible.

*Hexa-methylène-tetramine* was the drug at first hit upon, and whether when taken by mouth, absorbed and excreted into the urine, it liberates any formaldehyde recognisable as such is now not a point under discussion; it is admitted to increase remarkably the acidity of the urine, not necessarily by removal or destruction of bacteria, for in their absence a neutral alkaline healthy urine will be made strongly acid. This compound has been given the proprietary title of *urotropine*, but it has been given other less well known names, cystogen, cystamine, aminoform. Shortly after urotropine had acquired some vogue, a new compound of almost the same nature was suggested in the form of the anhydromethylene citrate of the first, which was called by the manufacturers *Helmitol*. For this it claimed the property of giving off formaldehyde more rapidly and in greater quantities than does urotropine; it is said moreover to have analgesic and sedative effects when the bladder wall and urethra are inflamed. Quite recently a third compound formed by the combination of Hexa-methylene-tetramine and resorcin has been advocated under the name "*Hetralin*," for which is claimed a more rapid liberation of formaldehyde and greater bactericidal powers *in vitro* than either urotropine or helmitol.

The choice between these drugs is difficult to base on any *a priori* grounds; their individual effects in particular cases seem to be the only sure guide. It is safe to assume that any compound of hexa-methylene-tetramine will show some power of increasing the acid reaction of the urine, but what part the additions to the formula play is quite uncertain; the peculiar properties appear to belong to the tetramine group, for if the diamine group is substituted the well known synthetic aperient "Purgen" is formed (Hexa-methylene-diamine). Probably the discussion leads eventually in the direction of the very intricate problem of excretion of amido-acids. The therapeutical uses of the whole group may be considered together, for the superiority of one over the other is not definitely proved in any particular condition.

*Cystitis.*—The best results are obtained in the case of ammoniacal urine depending on retention, where, as soon as the alkaline reaction is altered to an acid, the pus and phosphaturia will, as a rule, rapidly disappear. In tubercular ulceration of the bladder so favourable a termination cannot be looked for, the reaction of the urine is frequently not alkaline, and it cannot be demonstrated that the administration of Hexa-methylene-tetramine produces any specific effect upon bacilli, which are lodged deeply in the mucous membrane; if in addition to the tubercular disease a secondary infection is present, producing alkaline urine and phosphaturia, considerable improvement in the state of the urine may be procured.

*Prophylactic Use.*—In cases of spinal disease, enlarged prostate, or operations about the genito-urinary organs where catheterisation is likely to be required for any length of time, the risk of cystitis may be very greatly diminished by the daily administration of small doses while the urine is quite healthy.

With this precaution an occasional over-distension of the bladder is an accident less likely to be followed by the otherwise inevitable cystitis.

*Typhoid Fever.*—It is now recognised that potent as the fæces are in conveying the infection of typhoid fever the urine is no less so, and the typhoid bacillus may persist in the urine long after the patient is convalescent. Urotropine and its allies are particularly valuable as a means of destroying the bacillus and preventing the further dissemination of the disease. During the acute stages of the fever, when the urine becomes turbid and alkaline from cystitis or so-called typhoid bacilluria, these drugs will act rapidly and reliably in clearing up the condition. In fact, in bacilluria of all sorts (except tubercular) we find in urotropine a valuable and trustworthy remedy.

*Pyelitis.*—Since the cause of pyelitis is as a rule either renal calculus or tuberculosis of the kidney, it need not be expected that the cause of the disease will be removed by these means, but the state of the urine can be very materially improved, by reducing the suppuration and diminishing the risk of secondary infections. Under these circumstances the patient suffering from tubercular disease is put into a more favourable position for combating the malady, and by his own powers of resistance possibly recovering from it.

*Dosage.*—The doses recommended of urotropine and helmitol are 5 to 15 grs. The drugs are readily soluble in water, and the more concentrated the dose the less efficacious is it likely to prove. Five grains of urotropine given three times a day in a half or whole tumbler of water will probably give better results than 10 or 15 grs. in an ounce of water. In moderate doses no toxic or untoward effects are known to occur, except nausea if given on an empty stomach.

